# Supplementary material for: Structural insights into the peptide selectivity and activation of human neuromedin U receptors
Source: Nat Commun. 2022 Apr 19;13:2045. doi: 10.1038/s41467-022-29683-w (PMC9019041; doi:10.1038/s41467-022-29683-w)
Supplement: Supplementary file 1 — Supplementary Information [file 41467_2022_29683_MOESM1_ESM.pdf]

Supplementary Information

Structural insights into the peptide selectivity and activation of human neuromedin U receptors

Chongzhao You[1,2,5], Yumu Zhang[1,3,5], Peiyu Xu[1,2,5], Sijie Huang[1,3], Wanchao Yin[1], H. Eric Xu[1,2,3,†], Yi Jiang [1,3,4,†]

1. The CAS Key Laboratory of Receptor Research, Shanghai Institute of Materia Medica, Chinese Academy of Sciences, Shanghai 201203, China
2. University of Chinese Academy of Sciences, Beijing 100049, China
3. School of Life Science and Technology, ShanghaiTech University, Shanghai 201210, China
4. Lingang Laboratory, Shanghai 200031, China
5. These authors contributed equally: Chongzhao You, Yumu Zhang, and Peiyu Xu

[†] Correspondences: Y.J. (yijiang@simm.ac.cn) and H.E.X. (eric.xu@simm.ac.cn)

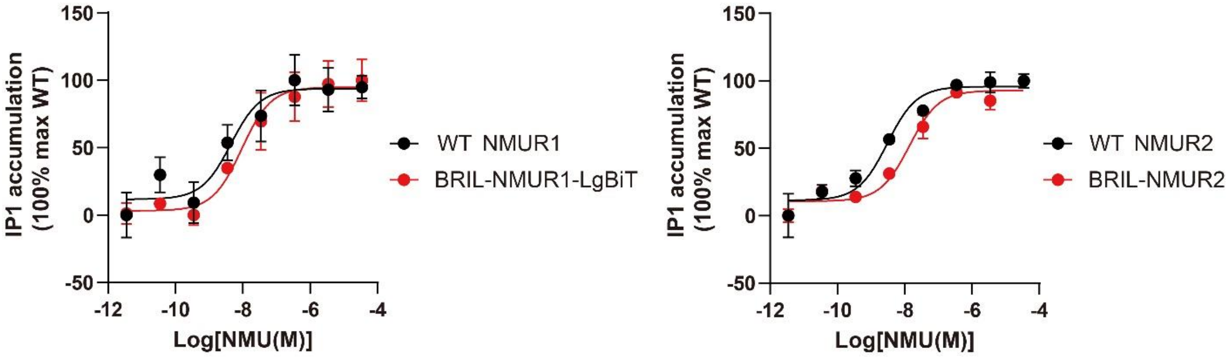

Supplementary Fig. 1 The comparison of wild-type or engineered NMU receptors response for NMU. BRIL-NMUR1-LgBiT and BRIL-NMUR2 are constructs used in cryo-EM structure determination. pEC_{50} values of NMU for the wild-type (WT) and modified NMUR1 are 8.35 ± 0.34 and 8.03 ± 0.27, respectively. pEC_{50} values of NMU for the WT and modified NMUR2 are 8.52 ± 0.17 and 7.86 ± 0.14, respectively. Each point represents mean ± S.E.M. from three independent experiments. Source data are provided as a Source Data file.

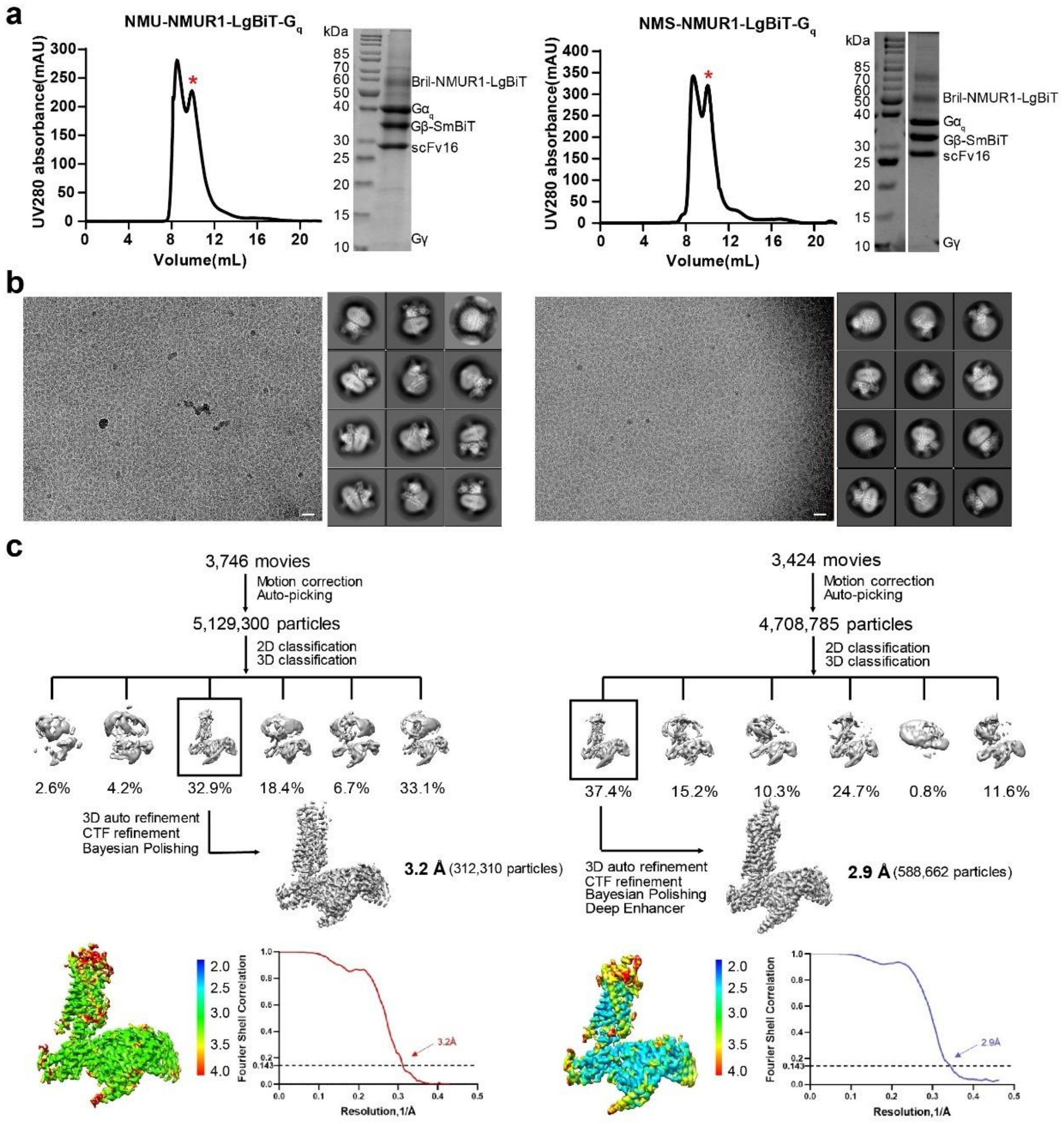

Supplementary Figure 2. NMU/NMS-NMUR1-G_q complexes purification and cryo-EM data processing. a Representative size-exclusion chromatography elution profile and SDS-PAGE analysis of NMU-NMUR1-G_q (left) and NMS-NMUR1-G_q complexes (right), respectively. Red asterisks refer to complex monomers. Source data are provided as a Source Data file. The cryo-EM sample preparation and data collection were performed once. **b** Cryo-EM micrographs and representative 2D average classes of both complexes are shown. Scale bar, 50 nm. **c** Flowchart of cryo-EM data processing, cryo-EM maps (colored by local resolution (Å) calculated using the ResMap package), and "Gold-standard" FSC curves.

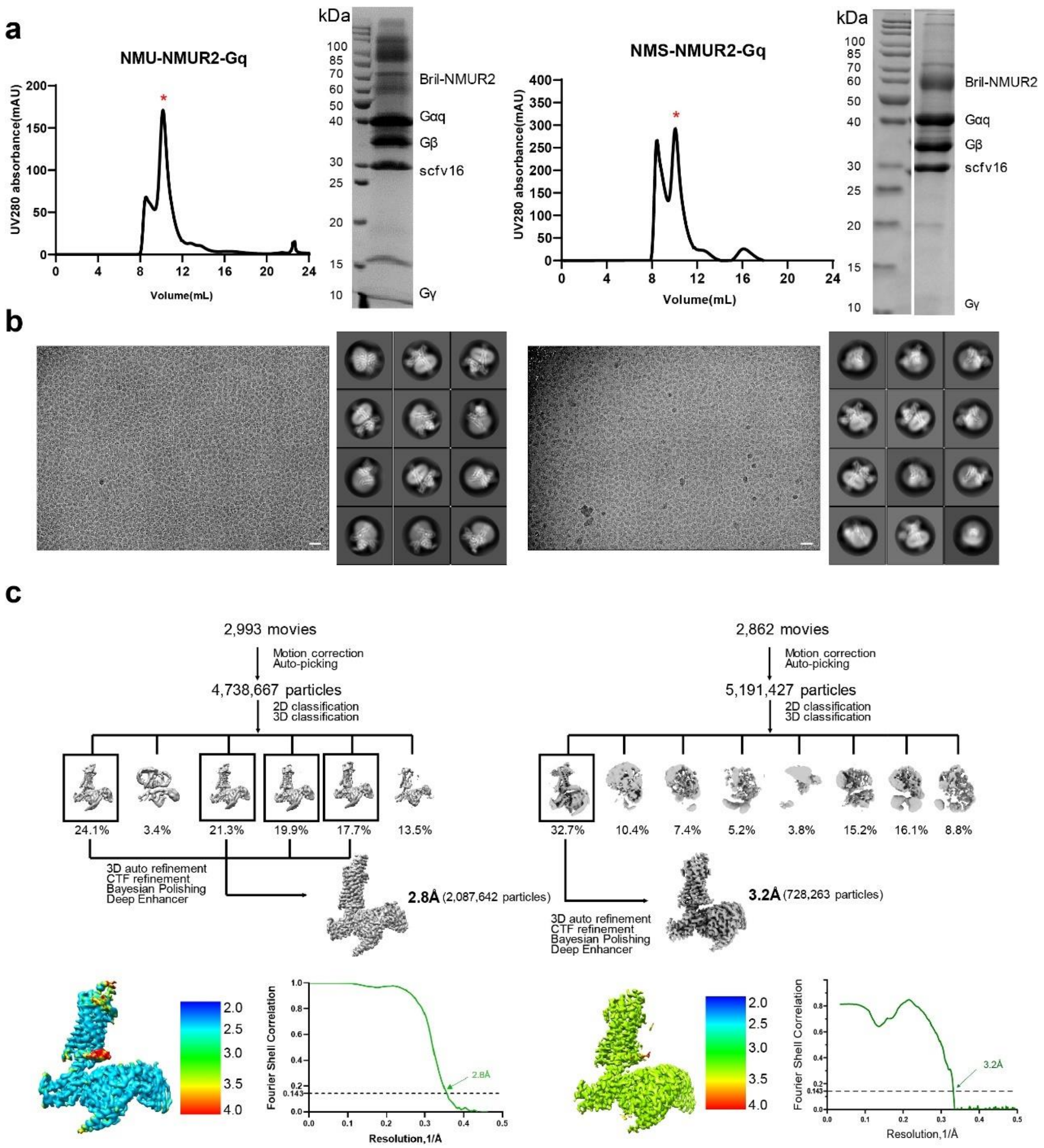

Supplementary Figure 3. NMU/NMS-NMUR2-G$_q$ complexes purification and cryo-EM data processing. a Representative size-exclusion chromatography elution profile and SDS-PAGE analysis of NMU-NMUR2-G$_q$ (left) and NMS-NMUR2-G$_q$ (right) complexes, respectively. Source data are provided as a Source Data file. The cryo-EM sample preparation and data collection were performed once. **b** Cryo-EM micrographs and representative 2D average classes of both complexes are shown. Scale bar, 50 nm. Red asterisks refer to complex monomer. **c** Flowchart of cryo-EM data processing, cryo-EM maps (colored by local resolution (Å) calculated using the Resmap package), and "Gold-standard" FSC curves.

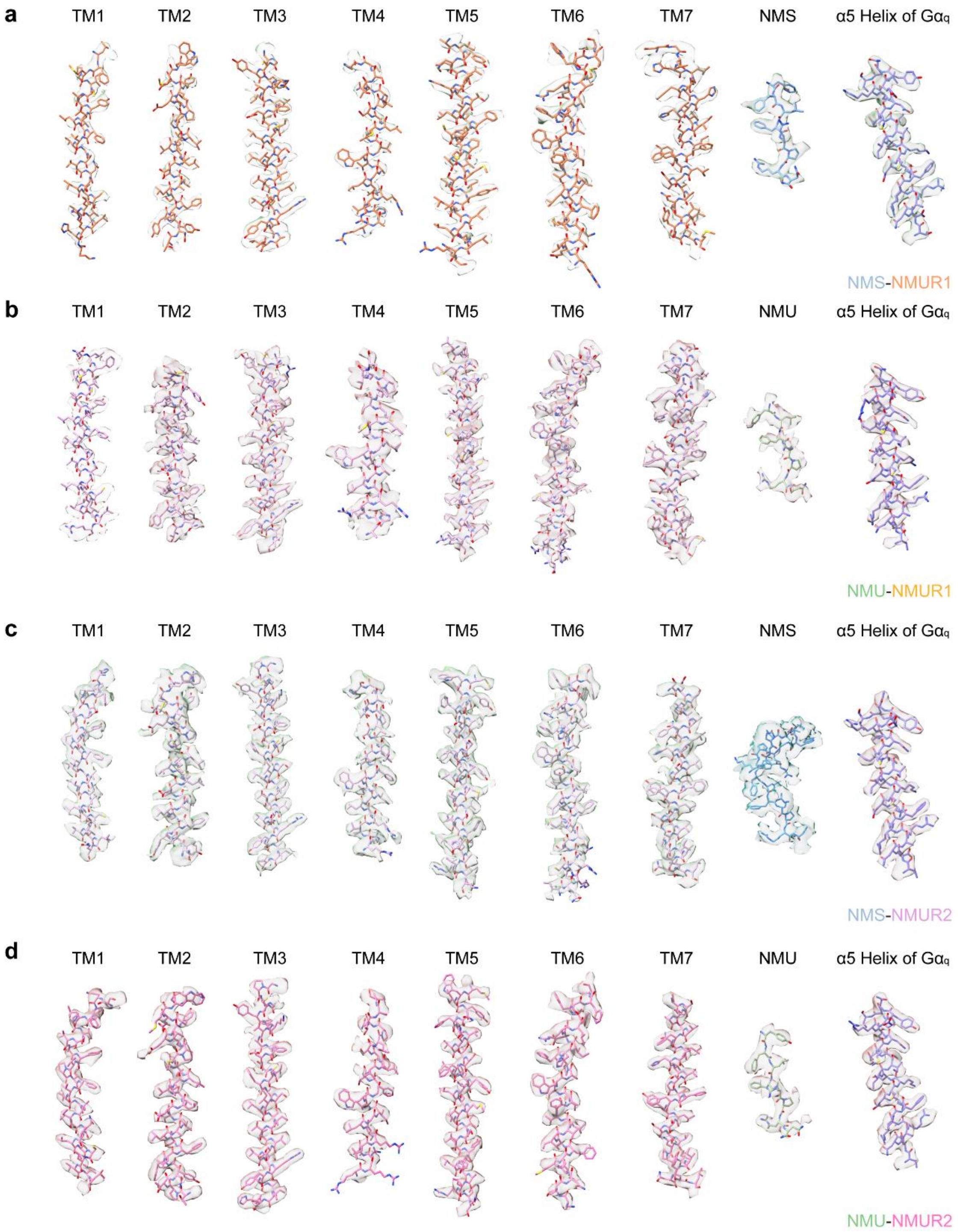

Supplementary Figure 4. Representative cryo-EM density maps of the NMU/NMS-NMUR1-G_q and NMU/NMS-NMUR2-G_q complexes. Cryo-EM density maps of the seven transmembrane (TM) helices, α5 helix of $G\alpha_q$, and corresponding peptides for NMS-bound NMUR1 (**a**), NMU-bound NMUR1 (**b**), NMS-bound NMUR2 (**c**), and NMU-bound NMUR2 (**d**) were shown. Colors are shown as indicated.

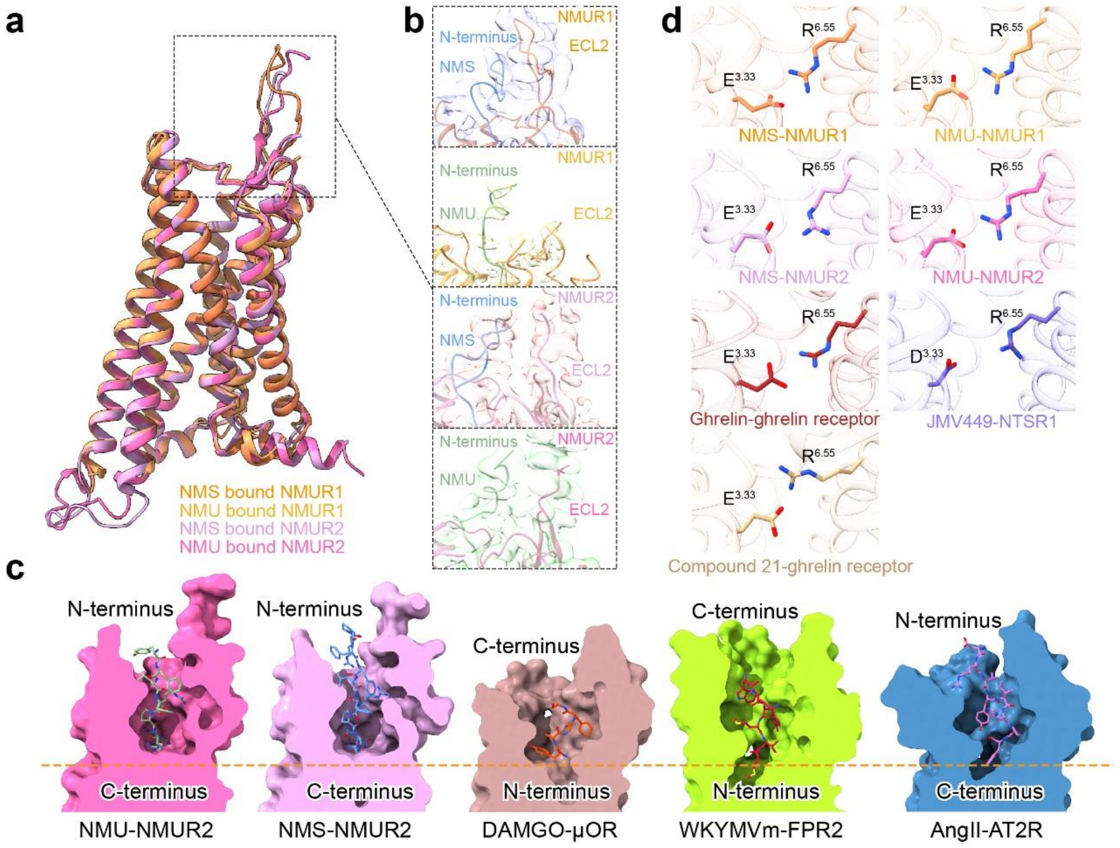

Supplementary Figure 5. Structural comparison of active NMURs with other peptide-bound class A GPCRs. a Structural superposition of four NMURs bound to NMU and NMS, respectively. **b** The possible interaction between N-termini of NMU/NMS and ECL2 of receptors. Ambiguous EM densities of the N-termini of peptides can be observed in these four complexes. **c** Structural comparison of the overall peptide-binding pocket of NMUR2 with other GPCRs solved to date. The orange dashed line refers to the bottom of ligand binding pockets of NMUR2. μOR, Mu Opioid Receptor (PDB 6DDE); FPR2, Formyl peptide receptor 2 (PDB 6OMM); AT2R, Angiotensin II type 2 receptor (PDB 6JOD). **d** The conserved salt bridge formed between E/D$^{3.33}$ and R$^{6.55}$. Colors are shown as indicated. Ghrelin receptor (ghrelin-bound, PDB 7F9Y); NTSR1, Neurotensin receptor type 1 (PDB 6OS9); Ghrelin receptor (compound 21-bound, PDB 6KO5).

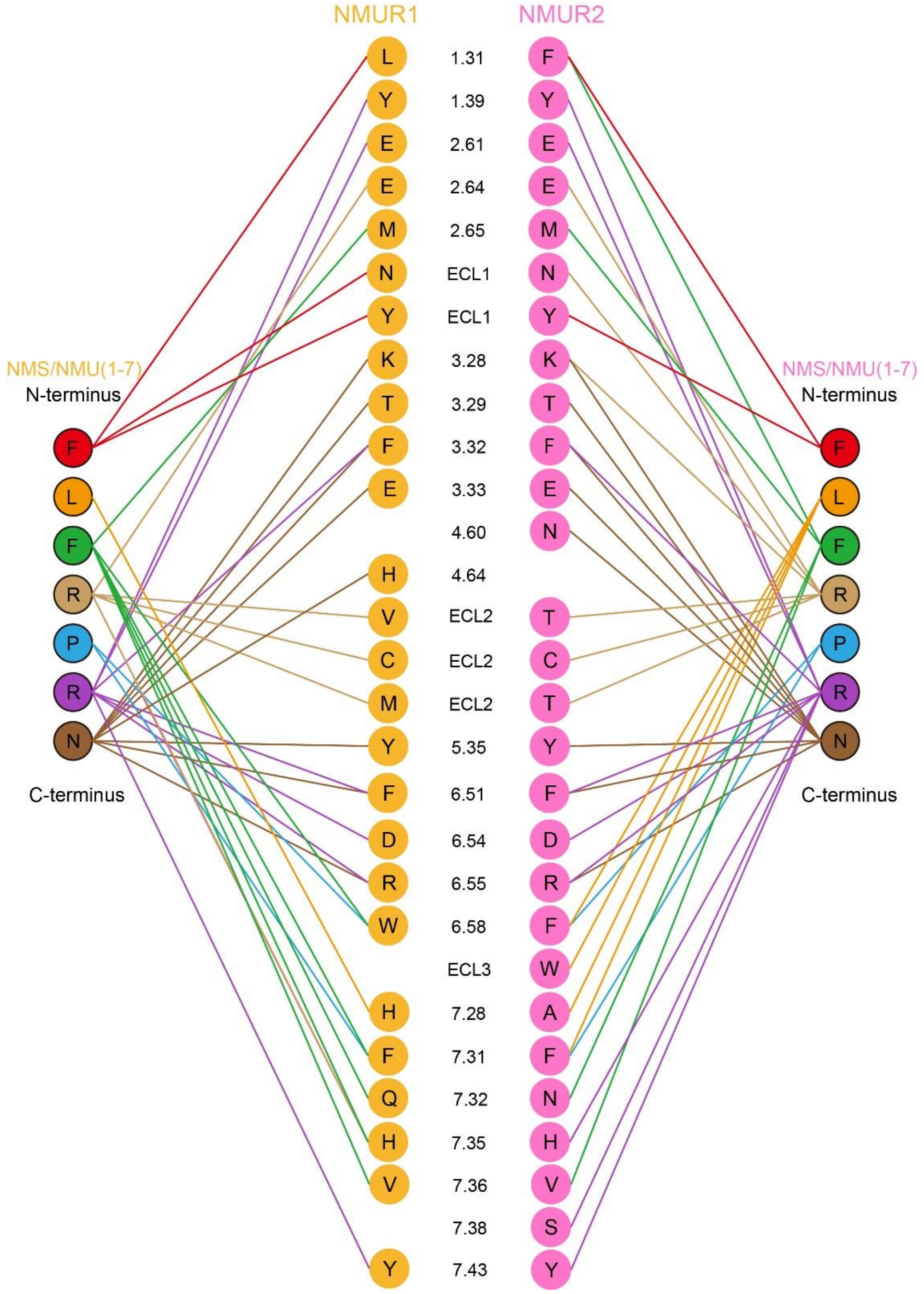

Supplementary Figure 6. Representative peptide-receptor interaction networks of NMU/NMS-NMUR1-G_q and NMU/NMS-NMUR2-G_q complexes. Colors are shown as indicated.

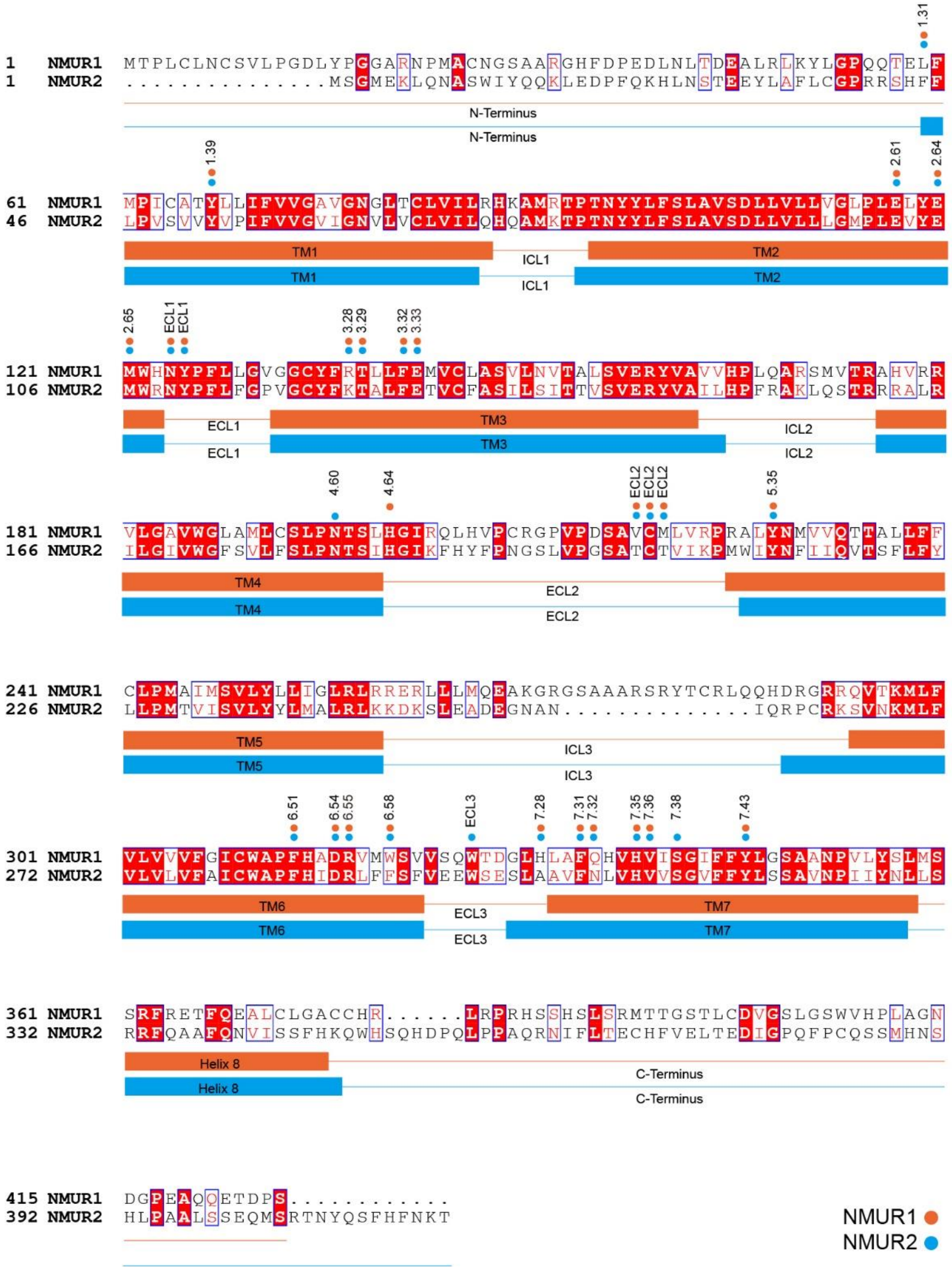

Supplementary Figure 7. Sequence alignment of the NMUR subfamily. The sequence alignment of NMUR1 and NMUR2 was generated by CLUSTALW (https://www.genome.jp/tools-bin/clustalw) and ESPript 3.0 (https://espript.ibcp.fr/ESPript/cgi-bin/ESPript.cgi). α-helices for NMUR1 and NMUR2 are shown as columns underneath the sequence. Orange dots represent the binding-pocket residues of NMUR1 bound to NMU/NMS. Blue dots represent the binding pocket residues of NMUR2 bound to NMU/NMS.

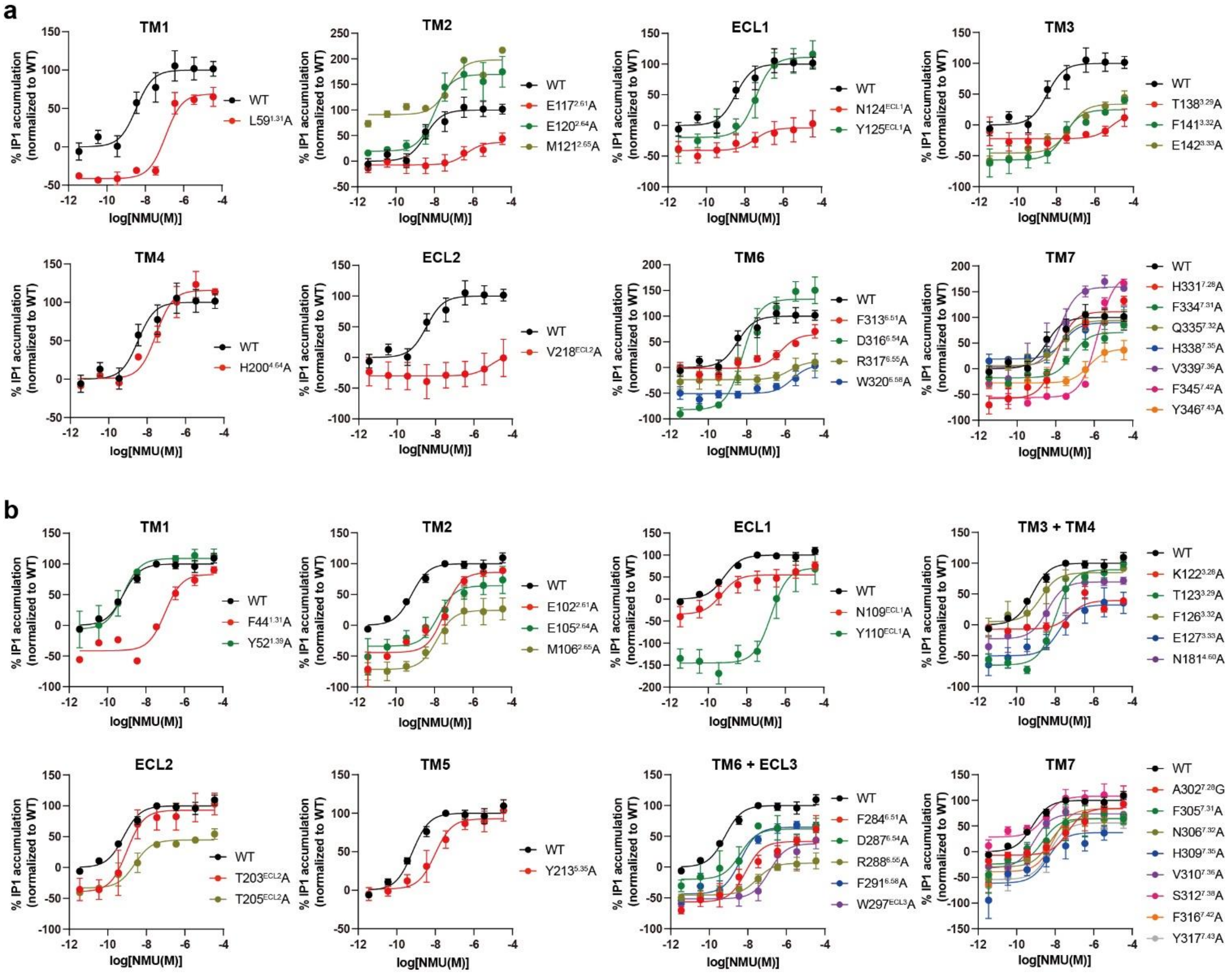

Supplementary Figure 8. Effects of mutation in the ligand-binding pocket of NMURs on IP-One accumulation. AD293 cells were transfected with wild-type (WT), NMUR1, or NMUR2 mutant constructs. Intracellular IP1 accumulation signals of WT and NMUR1 (**a**) and NMUR2 mutants (**b**) were monitored after stimulation with NMU. Each point represents mean ± S.E.M. from three independent experiments. The dataset links to Supplementary Fig. 9 and Supplementary Table 2. Source data are provided as a Source Data file.

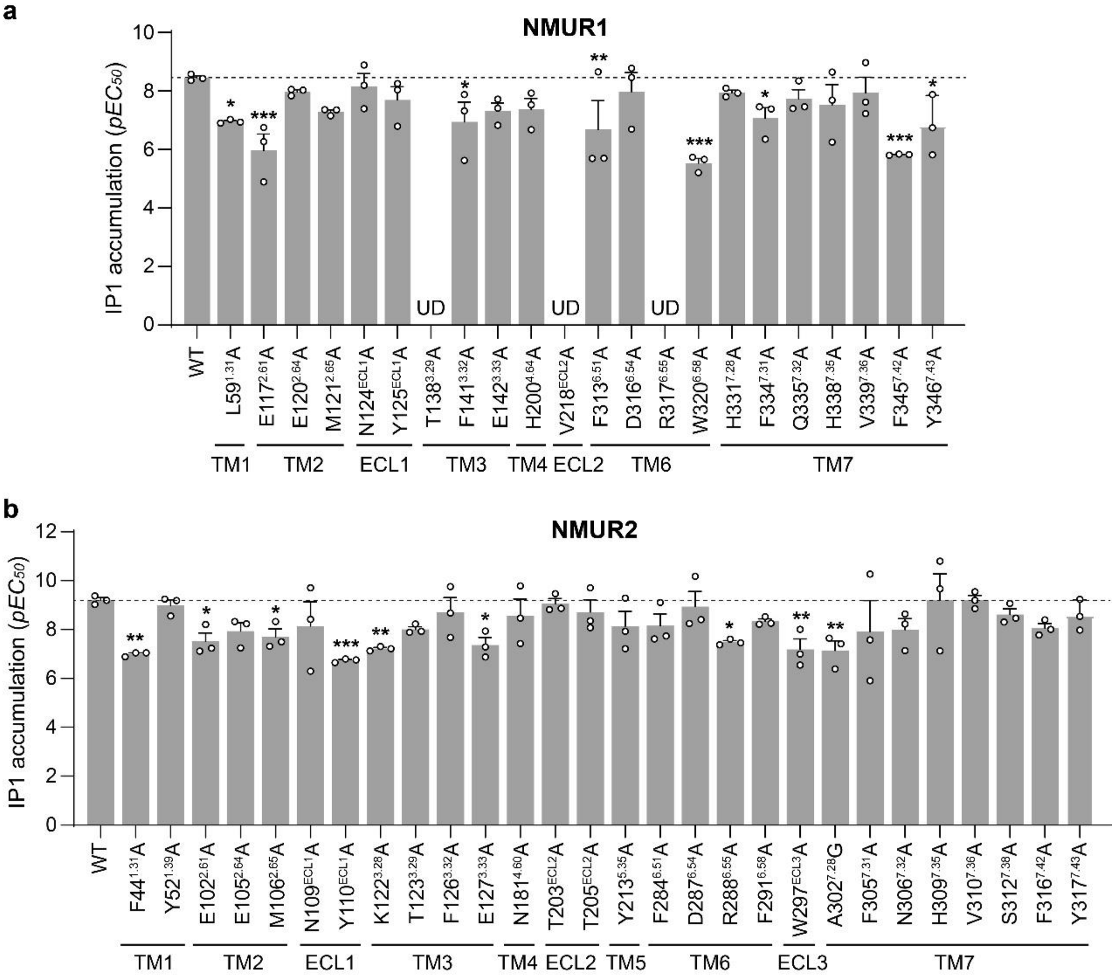

Supplementary Figure 9. Alanine mutagenesis analysis of peptide-binding pocket of NMURs. The IP-One assay was performed to evaluate the effects of NMU on the G_q-coupling activity of NMUR1 (**a**) and NMUR2 (**b**) mutants. Data were shown as mean pEC_{50} ± S.E.M. from three independent experiments in triplicate (n=3). The significance was determined with two-side, one-way ANOVA with Tukey's test. $^{*}P < 0.05$, $^{**}P < 0.01$, $^{***}P<0.001$ *vs.* wild-type (WT) receptor. UD, Undetectable. The dataset links to Supplementary Fig. 8 and Supplementary Table 2. Source data are provided as a Source Data file.

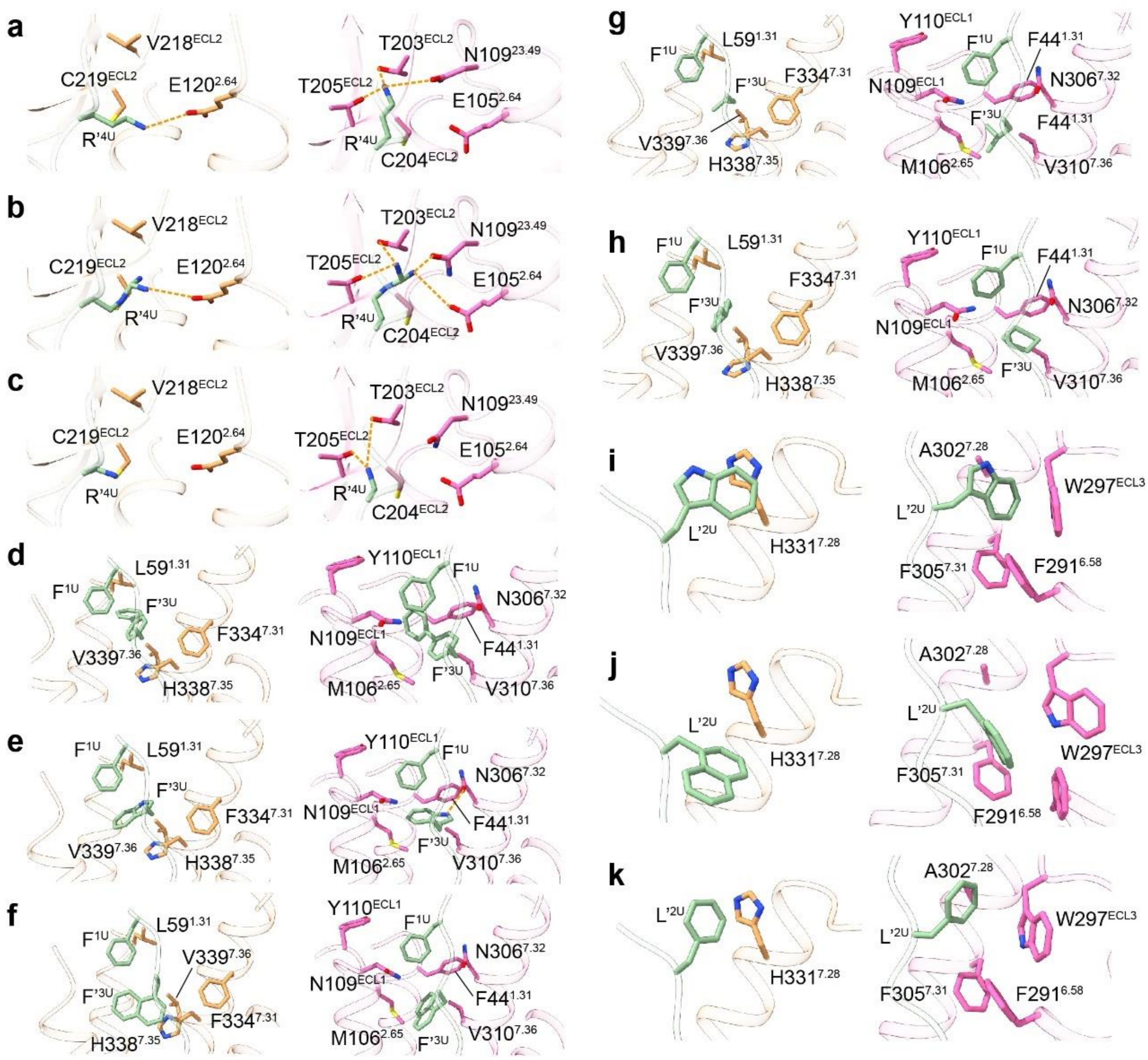

Supplementary Figure 10. Molecular docking analysis of selected NMU derivatives for NMUR1 and NMUR2. **a-c** Detailed interaction of various modified R^4 of NMU with residues in NMUR1 (Left) and NMUR2 (Right). (**a**) the guanidinium group was replaced by amino, (**b**) the side chain was shortened by one carbon atom, (**c**) guanidine replacement by amino and side chain shortened by two carbon atoms. **d-h** Detailed interaction of various modified F^4 of NMU with residues in NMUR1 (Left) and NMUR2 (Right). Biphenyl (**d**), indolyl (**e**), naphthyl (**f**), isopropyl (**g**) and cyclohexyl (**h**) substitutions were introduced, respectively. **i-k** Detailed interaction of various modified L^2 of NMU with residues in NMUR1 (Left) and NMUR2 (Right). Indolyl (**i**), naphthyl (**j**), and naphthyl (**k**) replacement were applied, respectively. Non-standard residues were generated by Discovery Studio 2016. The structures encountered a minimization process in Schrödinger Maestro. Colors are shown as indicated. Hydrogen bonds and salt bridges are depicted as orange dashed lines.

a

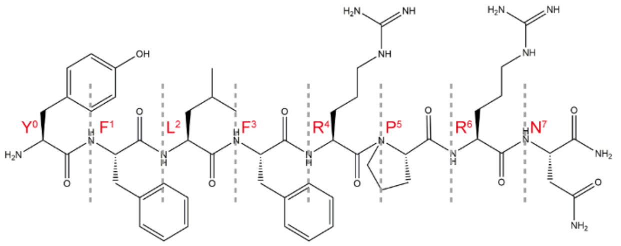

NMU-8 (Lead peptide)
EC_{50}(NMUR1)= 0.11 nM
EC_{50}(NMUR2)= 0.3 nM

b

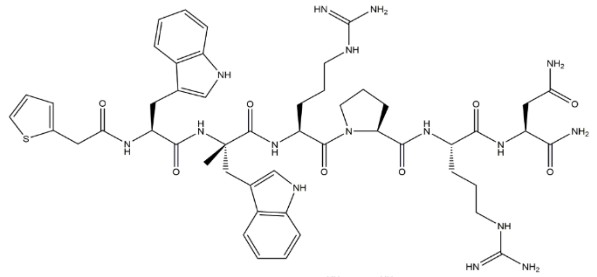

CPN-267
EC_{50}(NMUR1)= 0.25 nM
EC_{50}(NMUR2)= below 10^{-7} M

c

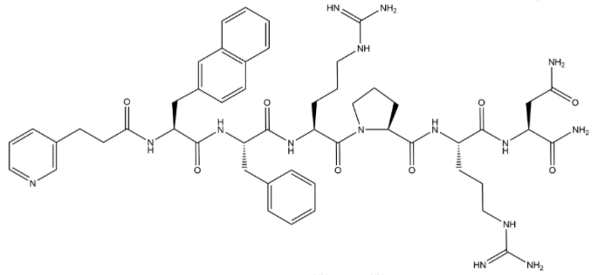

compound 8d (PMID:24999562)
EC_{50}(NMUR1)= 5.1 nM
EC_{50}(NMUR2)= 249 nM

d

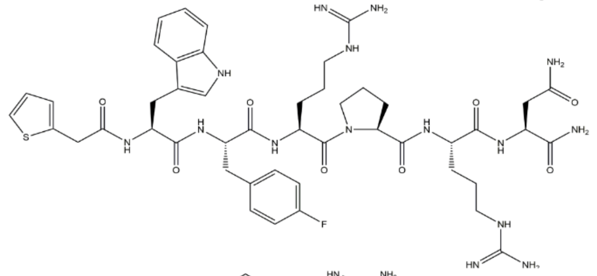

compound 5d (PMID:25815150)
EC_{50}(NMUR1)= 0.083 nM
EC_{50}(NMUR2)= 2.6 nM

e

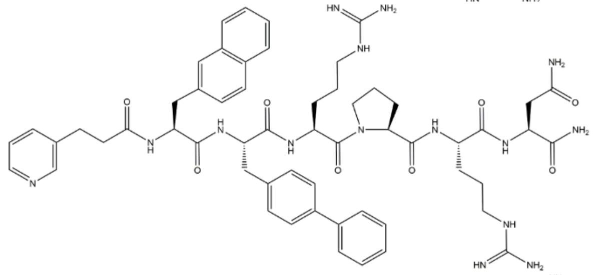

CPN-223
EC_{50}(NMUR1)= 3.2 nM
EC_{50}(NMUR2)= >1000 nM

f

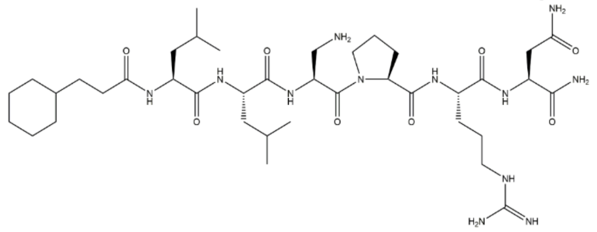

compound 6b (PMID:24999562)
EC_{50}(NMUR1)= >1000 nM
EC_{50}(NMUR2)= 6.6 nM

Supplementary Figure 11. Summary of reported NMUR1 and NMUR2 selective agonists derived from NMU-8.

Supplementary Table 1. Cryo-EM data collection, model refinement and validation statistics.

	NMU-NMUR1-G_q (EMD-32313) (PDB 7W53)	NMS-NMUR1-G_q (EMD-32315) (PDB 7W56)	NMU-NMUR2-G_q (EMD-32314) (PDB 7W55)	NMS-NMUR2-G_q (EMD-32316) (PDB 7W57)
**Data collection and processing**				
Magnification	64,000	64,000	64,000	64,000
Voltage (kV)	300	300	300	300
Electron exposure (e–/Å^2)	61.8	61.8	61.8	61.8
Defocus range (μm)	-1.0~-3.0	-1.0~-3.0	-1.0~-3.0	-1.0~-3.0
Pixel size (Å)	1.08	1.08	1.08	1.08
Symmetry imposed	C1	C1	C1	C1
Initial particle images (no.)	5,129,300	4,708,785	4,738,667	5,191,427
Final particle images (no.)	312,310	588,662	2,087,642	728,263
Map resolution (Å)	3.2	2.9	2.8	3.2
FSC threshold	0.143	0.143	0.143	0.143
Map resolution range (Å)	2-4	2-4	2-4	2-4
**Refinement**				
Initial model used (PDB code)				
Model resolution (Å)	3.3	3.2	3.1	3.1
FSC threshold	0.5	0.5	0.5	0.5
Model resolution range (Å)	50-3.3	50-3.2	50-3.1	50-3.1
Map sharpening *B* factor (Å^2)	-100	-77.9806	-199.894	-172.943
Model composition				
Non-hydrogen atoms	8709	8880	9220	9150
Protein residues	1109	1145	1167	1167
Ligands	-	-	-	-
B factors (Å^2)				
Protein	51.43	61.24	54.33	45.26
Ligand	-	-	-	-
R.m.s. deviations				
Bond lengths (Å)	0.005	0.007	0.006	0.004
Bond angles (°)	0.650	0.665	1.021	0.581
Validation				
MolProbity score	1.51	1.45	1.29	1.25
Clashscore	5.02	5.76	4.80	4.25
Poor rotamers (%)	0.21	0.61	0.40	0.89
Ramachandran plot				
Favored (%)	96.39	97.24	97.82	97.82
Allowed (%)	3.61	2.76	2.18	2.18
Disallowed (%)	0.00	0.00	0.00	0.00

Supplementary Table 2. pEC_{50} values of NMU on NMUR1 and NMUR2 mutants. IP-One assay was performed to evaluate NMU-induced receptor activation. Data are presented as means ± S.E.M. of three independent experiments (n=3). All data were analyzed by two-side, one-way ANOVA with Tukey's test. $^{*}P$<0.05, $^{**}P$<0.01, $^{***}P$<0.001 *vs.* wild-type (WT). The dataset links to Supplementary Figs. 8 and 9. Source data are provided as a Source Data file.

BW Numbering	NMUR1				NMUR2			
	**Mutant**	**IP1 accumulation pEC_{50} ± S.E.M.**	***P* value (IP1)**	**Surface expression (%WT) ± S.E.M**	**Mutant**	**IP1 accumulation pEC_{50} ± S.E.M.**	***P* value (IP1)**	**Surface expression (%WT) ± S.E.M**
-	WT	8.45 ± 0.06	>0.9999	100	WT	9.20 ± 0.10	>0.9999	100
1.31	L59A	6.96 ± 0.04 *	0.0256	119 ± 11	F44A	6.99 ± 0.05 **	0.0027	144 ± 21
1.39	NT	NT	NT	NT	Y52A	9.00 ± 0.22	0.7742	114 ± 17
2.61	E117A	5.97 ± 0.56 ***	0.0004	44 ± 3	E102A	7.51 ± 0.35 *	0.0200	69 ± 4
2.64	E120A	7.97 ± 0.07	0.4573	78 ± 3	E105A	7.93 ± 0.35	0.0776	192 ± 4
2.65	M121A	7.29 ± 0.07	0.0774	151 ± 14	M106A	7.71 ± 0.31 *	0.0387	240 ± 4
ECL1	N124A	8.16 ± 0.43	0.6473	80 ± 1	N109A	8.14 ± 0.99	0.1381	171 ± 8
ECL1	Y125A	7.70 ± 0.45	0.2437	87 ± 3	Y110A	6.73 ± 0.05 ***	0.0009	45 ± 2
3.28	NT	NT	NT	NT	K122A	7.21 ± 0.05 **	0.0066	42 ± 1
3.29	T138A	UD	<0.0001	110 ± 27	T123A	8.01 ± 0.11	0.0966	250 ± 27
3.32	F141A	6.94 ± 0.67 *	0.0237	149 ± 12	F126A	8.70 ± 0.60	0.4835	113 ± 9
3.33	E142A	7.32 ± 0.26	0.0856	96 ± 11	E127A	7.36 ± 0.31 *	0.0116	193 ± 19
4.60	NT	NT	NT	NT	N181A	8.56 ± 0.68	0.3685	214 ± 55
4.64	H200A	7.38 ± 0.37	0.1019	95 ± 18	NT	NT	NT	NT
ECL2	V218A	UD	<0.0001	142 ± 7	T203A	9.05 ± 0.21	0.8339	172 ± 50
ECL2	NT	NT	NT	NT	T205A	8.71 ± 0.50	0.4918	67 ± 7
5.35	NT	NT	NT	NT	Y213A	8.14 ± 0.61	0.1379	121 ± 16
6.51	F313A	6.68 ± 0.98 **	0.0088	97 ± 8	F284A	8.15 ± 0.48	0.1434	154 ± 13
6.54	D316A	7.98 ± 0.65	0.4616	156 ± 53	D287A	8.94 ± 0.62	0.7127	62 ± 5
6.55	R317A	UD	<0.0001	74± 4	R288A	7.48 ± 0.07 *	0.0177	130 ± 1
6.58	W320A	5.54 ± 0.16 ***	<0.0001	81 ± 11	F291A	8.33 ± 0.10	0.2231	189 ± 14
ECL3	NT	NT	NT	NT	W297A	7.18 ± 0.43 **	0.0058	76 ± 4
7.28	H331A	7.94 ± 0.08	0.4288	111 ± 30	A302G	7.15 ± 0.39 **	0.0051	172 ± 50
7.31	F334A	7.07 ± 0.36 *	0.0369	113 ± 1	F305A	7.91 ± 1.27	0.0731	87 ± 3
7.32	Q335A	7.74 ± 0.31	0.2712	124 ± 12	N306A	8.00 ± 0.45	0.0932	167 ± 10
7.35	H338A	7.53 ± 0.69	0.1578	222 ± 22	H309A	9.19 ± 1.08	0.9932	174 ± 25
7.36	V339A	7.94 ± 0.53	0.4274	97 ± 3	V310A	9.20 ± 0.20	0.9970	83 ± 6
7.38	NT	NT	NT	NT	S312A	8.61 ± 0.23	0.4078	140 ± 8
7.42	F345A	5.83 ± 0.01 ***	0.0002	121 ± 4	F316A	8.06 ± 0.18	0.1110	112 ± 7
7.43	Y346A	6.81 ± 0.60 *	0.0146	98 ± 4	Y317A	8.58 ± 0.36	0.3829	126 ± 6

BW numbering, Ballesteros & Weinstein numbering, a generic GPCR residue numbering scheme; UD, Undetectable; NT, Not tested.
