# Supplementary material for: Structural insights into the peptide selectivity and activation of human neuromedin U receptors
Source: Nat Commun. 2022 Apr 19;13:2045. doi: 10.1038/s41467-022-29683-w (PMC9019041; doi:10.1038/s41467-022-29683-w)
Supplement: Supplementary file 2 — Reporting Summary [file 41467_2022_29683_MOESM2_ESM.pdf]

nature portfolio

Corresponding author(s): Yi Jiang, H. Eric Xu

Last updated by author(s): Mar 4, 2022

Reporting Summary

Nature Portfolio wishes to improve the reproducibility of the work that we publish. This form provides structure for consistency and transparency in reporting. For further information on Nature Portfolio policies, see our Editorial Policies and the Editorial Policy Checklist.

Statistics

For all statistical analyses, confirm that the following items are present in the figure legend, table legend, main text, or Methods section.

n/a	Confirmed	
☐	☒	The exact sample size (*n*) for each experimental group/condition, given as a discrete number and unit of measurement
☐	☒	A statement on whether measurements were taken from distinct samples or whether the same sample was measured repeatedly
☐	☒	The statistical test(s) used AND whether they are one- or two-sided<br>*Only common tests should be described solely by name; describe more complex techniques in the Methods section.*
☒	☐	A description of all covariates tested
☒	☐	A description of any assumptions or corrections, such as tests of normality and adjustment for multiple comparisons
☐	☒	A full description of the statistical parameters including central tendency (e.g. means) or other basic estimates (e.g. regression coefficient) AND variation (e.g. standard deviation) or associated estimates of uncertainty (e.g. confidence intervals)
☐	☒	For null hypothesis testing, the test statistic (e.g. *F*, *t*, *r*) with confidence intervals, effect sizes, degrees of freedom and *P* value noted<br>*Give P values as exact values whenever suitable.*
☐	☒	For Bayesian analysis, information on the choice of priors and Markov chain Monte Carlo settings
☒	☐	For hierarchical and complex designs, identification of the appropriate level for tests and full reporting of outcomes
☒	☐	Estimates of effect sizes (e.g. Cohen's *d*, Pearson's *r*), indicating how they were calculated

Our web collection on statistics for biologists contains articles on many of the points above.

Software and code

Policy information about availability of computer code

Data collection	Automated data collection on the Titan Krios was performed using serialEM 3.7.11.
Data analysis	The following software was used in this study: PyMol 2.5, MotionCor2.1, Ctffind4, Relion 3.1, Resmap 1.1.4, UCSF Chimera v1.14, UCSF ChimeraX 1.1, Phenix 1.18.2, Coot 0.9 EL, ISODLE1.1, Prism8, Discovery Studio 2016, Schrödinger Maestro 11.1, CLUSTALW 2.1, ESpript 3.0.

For manuscripts utilizing custom algorithms or software that are central to the research but not yet described in published literature, software must be made available to editors and reviewers. We strongly encourage code deposition in a community repository (e.g. GitHub). See the Nature Portfolio guidelines for submitting code & software for further information.

Data

Policy information about availability of data

All manuscripts must include a data availability statement. This statement should provide the following information, where applicable:

- Accession codes, unique identifiers, or web links for publicly available datasets
- A description of any restrictions on data availability
- For clinical datasets or third party data, please ensure that the statement adheres to our policy

The atomic coordinates and the electron microscopy maps have been deposited in the Protein Data Bank (PDB) under accession number 7W53, 7W55, 7W56, and 7W57, as well as Electron Microscopy Data Bank (EMDB) accession number EMD-32313, EMD-32314, EMD-32315, and EMD-32316 for the NMU-NMUR1-Gq-scFv16, NMU-NMUR2-Gq-scFv16, NMS-NMUR1-Gq-scFv16, and NMS-NMUR2-Gq-scFv16 complexes, respectively. Source data are provided with this paper.

Field-specific reporting

Please select the one below that is the best fit for your research. If you are not sure, read the appropriate sections before making your selection.

☒ Life sciences ☐ Behavioural & social sciences ☐ Ecological, evolutionary & environmental sciences

For a reference copy of the document with all sections, see nature.com/documents/nr-reporting-summary-flat.pdf

Life sciences study design

All studies must disclose on these points even when the disclosure is negative.

Sample size	For cryo-EM data, images were collected until the resolution and 3D reconstruction converges. For all the functional assay, no statistical approaches were used to predetermine the sample size. We use a sample size of three independent experiments, commonly exploited by researchers in this field.
Data exclusions	No data were excluded.
Replication	Each experimental findings were reliably reproduced within one month.
Randomization	Randomization was not relevant to this study, as the data were collected automatically and did not involve choosing.
Blinding	Blinding was not relevant to this study, since all the data were collected automatically.

Reporting for specific materials, systems and methods

We require information from authors about some types of materials, experimental systems and methods used in many studies. Here, indicate whether each material, system or method listed is relevant to your study. If you are not sure if a list item applies to your research, read the appropriate section before selecting a response.

Materials & experimental systems

n/a	Involved in the study	
☒	☐	Antibodies
☐	☒	Eukaryotic cell lines
☒	☐	Palaeontology and archaeology
☒	☐	Animals and other organisms
☒	☐	Human research participants
☒	☐	Clinical data
☒	☐	Dual use research of concern

Methods

n/a	Involved in the study	
☒	☐	ChIP-seq
☒	☐	Flow cytometry
☒	☐	MRI-based neuroimaging

Eukaryotic cell lines

Policy information about cell lines

Cell line source(s)	High Five Cells (ThermoFisher)<br>AD293 cells (Agilent)
Authentication	Used as expression stains only, independent verification after purchase not required.
Mycoplasma contamination	Cell lines were tested and free from mycoplasma contamination.
Commonly misidentified lines (See ICLAC register)	No commonly misidentified cell lines were used.
